# Endothelium-Derived Extracellular Vesicles Associate with Poor Prognosis in Metastatic Colorectal Cancer

**DOI:** 10.3390/cells9122688

**Published:** 2020-12-15

**Authors:** Afroditi Nanou, Linda Mol, Frank A. W. Coumans, Miriam Koopman, Cornelis J. A. Punt, Leon W. M. M. Terstappen

**Affiliations:** 1 Department of Medical Cell BioPhysics, University of Twente, 7522ND Enschede, The Netherlands; f.a.w.coumans@utwnte.nl; 2 Netherlands Comprehensive Cancer Organization, 6533AA Nijmegen, The Netherlands; L.Mol@iknl.nl; 3 Department of Medical Oncology, University Medical Center Utrecht, 3584CS Utrecht, The Netherlands; m.koopman-6@umcutrecht.nl; 4 Julius Center for Health Sciences and Primary Care, Department of Epidemiology, University Medical Center Utrecht, 3584CG Utrecht, The Netherlands; C.J.A.Punt@umcutrecht.nl

**Keywords:** metastatic colorectal cancer (mCRC), endothelium-derived extracellular vesicles (edEVs), circulating endothelial cells (CECs), CellSearch, ACCEPT, CAIRO2

## Abstract

Elevated, tumor-derived extracellular vesicle (tdEV) and circulating tumor cell (CTC) loads in metastatic cancer are associated with poor clinical outcome. Herein, we investigate whether endothelium-derived extracellular vesicles (edEVs) can be detected in the blood of metastatic colorectal cancer (mCRC) patients, and whether those vesicles associate with prognosis. The open-source ACCEPT (Automated CTC Classification, Enumeration, and Phenotyping) software was used to enumerate edEVs, tdEVs, and other objects from digitally stored CellSearch images acquired after CTC and circulating endothelial cell (CEC) enrichment from the blood of 395 mCRC patients before the initiation of a new therapy. Patients had participated in the prospective phase III CAIRO2 study. The presence of edEVs was found 5- to 10-fold higher than CECs. The hazard ratio (HR) (95% CI) of progression-free survival (PFS) for increased CTCs (≥3 in 7.5 mL), tdEVs (≥40 in 7.5 mL), and edEVs (≥287 in 4.0 mL.) was 1.4 (1.1–1.9), 2.0 (1.5–2.6), and 1.7 (1.2–2.5), respectively. The HR of Overall Survival (OS) for increased CTCs, tdEVs and edEVs was 2.2 (1.7–3.0), 2.7 (2.0–3.5), and 2.1 (1.5–2.8), respectively. There was no cut-off value for CECs, leading to a dichotomization of patients with a significant HR. Only tdEVs remained a significant predictor of OS in the final multivariable model.

## 1. Introduction

The necessity of non-invasive, disease-specific, and reliable biomarkers in different cancers is becoming urgent for the administration of targeted therapies and the evaluation of their efficacy in a timely fashion. In approximately 30% of advanced colorectal cancer patients, three or more circulating tumor cells (CTCs) in 7.5 mL of blood are detected, and these patients have a significantly shorter progression-free (PFS) and overall survival (OS) [1,2]. Therapy monitoring through increases or decreases in the number of CTCs is therefore only meaningful in these 30% of patients. Recently, we have shown that tumor-derived extracellular vesicles (tdEVs) are co-isolated with the CTCs and can be automatically enumerated in fluorescence images [3]. These tdEVs are present in approximately 20× higher frequencies compared to CTCs in patients with advanced prostate, breast, non-small-cell lung, and colorectal cancer, and tdEVs’ presence is also strongly associated with a poor clinical outcome [3,4,5]. The presence of circulating endothelial cells (CECs) in the blood of cancer patients has also been investigated, and has been found in higher frequencies in the blood of patients with advanced cancer compared to healthy donors [6]. The release of a portion of the detected endothelial cells can be attributed to their detachment from the blood vessel wall during the blood draw. As this varies between collected blood samples, it has a significant influence on the actual CEC counts and their reliability with regard to following disease processes [6]. We postulated that if endothelial cells produce extracellular vesicles in a similar way as tumor cells, we might be able to detect them and their count would most likely be more reliable, since their formation would be independent of the blood draw and the damage of the vasculature. For this study, we used a digitally stored set of fluorescence images generated from the blood samples of 395 advanced colorectal cancer patients receiving first-line chemotherapy and targeted agents before the initiation of a new therapy. The patients had participated in the phase III CAIRO2 study. The blood samples were processed with the CellSearch CTC kit for CTC and tdEV enumeration, as well as the CellSearch Endothelial Cell kit for CEC and edEV enumeration. The CTC and CEC data have been reported earlier [2,7]. We used the open-source ACCEPT (Automated CTC Classification, Enumeration, and Phenotyping) software to enumerate tdEVs and explore the presence of endothelium-derived extracellular vesicles (edEVs).

## 2. Materials and Methods

### 2.1. Patients

All patients included in the present analysis had participated in the prospective phase III CAIRO2 study (ClinicalTrials.gov identifier: NCT00208546) of the Dutch Colorectal Cancer Group [8]. All subjects had given their informed consent for inclusion before they participated in the study. The study was conducted in accordance with the Declaration of Helsinki, and the protocol was approved by the Committee on Research Involving Human Subjects Arnhem-Nijmegen (project identification code: CMO 2005/076).

Patients were randomly assigned to receive first-line treatment with capecitabine, oxaliplatin, and bevacizumab, or the same schedule with the addition of weekly cetuximab. Tumor response was assessed every 9 weeks using computed tomography (CT) imaging, and evaluated according to the Response Evaluation Criteria in Solid Tumors (RECIST) [9]. Details of the study have been reported previously [8,10].

### 2.2. Isolation of CTCs, tdEVs, CECs, and edEVs

The CellSearch CTC assay (Menarini Silicon Biosystems, Huntingdon Valley, PA, USA) was used to immunomagnetically isolate CTCs and tdEVs, based on their epithelial cell adhesion molecule (EpCAM) expression from 7.5 mL of blood collected in CellSave (Menarini Silicon Biosystems, Huntingdon Valley, PA, USA) tubes. The positively enriched objects were stained with the nuclear dye 4′,6-diamidino-2-phenylindole (DAPI), allophycocyanin-conjugated monoclonal antibodies against the leukocyte-specific cluster of differentiation 45 (CD45-APC), and phycoerythrin-conjugated monoclonal antibodies against cytokeratins 8, 18, and 19 (CK-PE). The suspension was placed in a cartridge contained within a magnet, as previously described [11].

The CellSearch Endothelial Cell assay (Menarini) was used to immunomagnetically enrich for CECs and edEVs expressing CD146 from 4.0 mL of CellSave blood. The positively enriched objects were stained with the nuclear dye DAPI and the fluorescently labeled monoclonal antibodies against CD45 (CD45-APC) and endoglin (CD105-PE). Image acquisition of both EpCAM- and CD146-enriched and stained objects within cartridges was performed on the CellTracks Analyzer II, a semi-automated fluorescence microscope equipped with computer-controlled X, Y, and Z stages, a 10× objective with 0.45 numerical aperture, a Mercury Arc lamp, a 12-bit charge-coupled device (CCD) camera, and filter cubes for DAPI, PE, APC, and fluorescein isothiocyanate (FITC). Typically, 175 images per fluorescence channel are acquired to cover the surface of the whole cartridge [11].

From this multicenter, phase III trial study, CTCs were enumerated before the initiation of therapy in 467 of the 755 advanced colorectal cancer patients and CECs in 473 of the 755 patients using the CellSearch system, and results have been reported earlier [2,7]. Fluorescence images of samples processed with the CTC and CEC kits were stored on digital versatile discs and transferred to hard disks in 450 of the 457 CTC samples and 395 of the 473 CEC samples. We re-analyzed the stored images of 395 patients for both CTC and CEC samples to identify the leukocytes, nucleated cells, and subpopulations of extracellular vesicles (EVs) co-isolated with both CTC and CEC kits.

### 2.3. Enumeration of tdEVs, edEVs, ldEVs, and Nucleated Cells by ACCEPT

For this study, the original CTC and CEC scores (manual counts) were used. To enumerate tdEVs, edEVs, leukocyte-derived extracellular vesicles (ldEVs), leukocytes, and nucleated cells, the digitally stored fluorescence image files were re-analyzed with the open-source Automated CTC Classification, Enumeration, and Phenotyping (ACCEPT) software (http://github.com/LeonieZ/ACCEPT), using the “Full Detection” processor. For the samples processed with the CEC kit, CD105+ leukocytes and CD105+ ldEVs were also identified and enumerated. The gates for each of the abovementioned populations in the samples processed with the CTC and CEC kits are provided in the Table S1.

### 2.4. Statistical Analysis

Statistical analysis was performed using the IBM SPSS platform for Windows, version 23.0 (SPSS Inc., Chicago, IL, United States) and MedCalc for Windows, version 18.0 (MedCalc Software, Ostend, Belgium). The primary objective was to assess the prognostic value of tdEVs and edEVs in mCRC patients. Eligible patients were assessable for these analyses if both a CTC and CEC sample was available prior to the start of a therapy. Patients were prospectively divided into patients with favorable and unfavorable CTC counts (<3 and ≥3, respectively). For tdEVs, the value of 40 was used as a cut-off, based on our previous results supporting the additional dichotomization of patients with favorable CTC counts using that value [5]. Overall survival (OS) was defined as the elapsed time in months between the baseline blood draw date and the date of death or last follow-up. Progression-free survival (PFS) was defined as the elapsed time in weeks between the baseline blood draw date and the date of progression or death, whichever occurred first. Patients alive at the end of the study or lost during the follow-up were censored. Median PFS and OS were defined by Kaplan–Meier survival curves. Kaplan–Meier survival curves of favorable and unfavorable groups were compared using a non-parametric log–rank test.

The open-source web application Cutoff Finder (http://molpath.charite.de/cutoff/) was used to calculate the hazard ratios (HRs) for PFS and OS, including 95% confidence intervals (CIs), over a wide range of cut-off values for CECs and edEVs of the 395 patients before the initiation of a new treatment. The optimal cut-off value of edEVs was defined as the point with the most significant split (minimum *p*-value, log–rank). Cutoff Finder uses the R code to provide optimization and visualization tools for cut-off determination [12].

Univariable Cox proportional hazards regression analysis was performed to determine the HR for PFS and OS, with 95% CI for each continuous and categorical variable. Continuous complete blood count (CBC)-based parameters, counts of CECs, CTCs, leukocytes, nucleated events, and EVs, isolated with the CEC and CTC kits, were log-transformed to improve the model fit. In case of the isolated events with the CTC and CEC kits, a constant value of 0.1 was added to deal with 0 values. A final multivariable Cox model was fit, including significant variables from the univariable Cox proportional hazards regression analysis. Due to correlation between some of the included variables, the final model was selected using backward stepwise elimination (*p_in_* = 0.05 and *p_out_* = 0.10).

The non-parametric, two-tailed Spearman’s Rho test was used to assess the strength of association between the available prognostic clinical parameters of the patients and the investigating biomarkers, namely CTCs, tdEVs, CECs, and edEVs. The same test was used to evaluate the strength of association between CBC-based parameters and the different classes of objects isolated with the CellSearch using the CTC and CEC kits. The non-parametric Wilcoxon signed ranks test was used to compare the distributions of matched leukocytes, ldEVs and nucleated events isolated with the different kits. Receiver operating characteristic (ROC) curves were used to assess the performance of a test using solely edEVs, CTCs, tdEVs, or a combination of edEVs with either CTCs or tdEVs. The classification variable was the shorter patient survival compared to the median OS of the patient cohort used. The areas under the curve (AUCs) were compared using the non-parametric DeLong approach [13].

## 3. Results

### 3.1. Identification of tdEVs, edEVs, ldEVs, Leukocytes, and Nucleated Cells by ACCEPT

**Figure 1:**
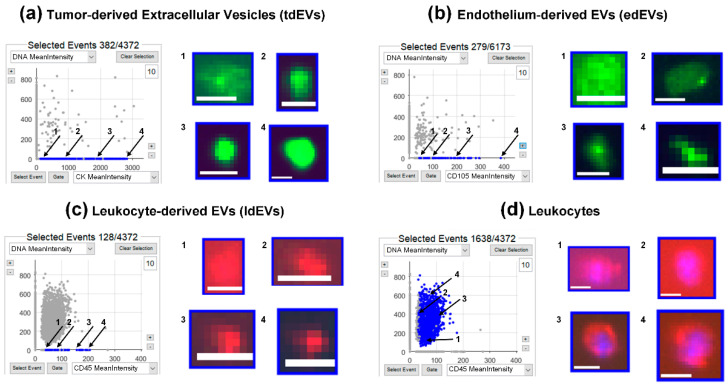
Examples of tumor-derived extracellular vesicles (tdEVs) (**a**), endothelium-derived extracellular vesicles (edEVs) (**b**), leukocyte-derived extracellular vesicles (ldEVs) (**c**), and leukocytes (**d**), isolated with the CellSearch system and identified by the ACCEPT (Automated CTC (circulating tumor cell) Classification, Enumeration, and Phenotyping) software. The dots corresponding to the objects that fall within the defined gates are depicted in blue in the respective scatter plots, whereas all other objects are depicted in grey. For each class, examples of four thumbnails with different fluorescence intensities (1–4) are shown. Cytokeratin (CK) (**a**) and endoglin (CD105) (**b**) are depicted in green, CD45 in red, and 4′,6-diamidino-2-phenylindole (DAPI) in blue (**c** and **d**). Scale bars indicate 6.4 μm.

Digitally stored fluorescence images, which were originated from the CellTracks Anayzer II, were analyzed by ACCEPT to identify tdEVs, edEVs, ldEVs, leukocytes, and nucleated cells, using the gates provided in Table S1. Figure 1 shows an example of a scatter plot for tdEVs (panel A), edEVs (panel B), ldEVs (panel C), and leukocytes (panel D). The ldEVs and leukocytes presented in this example were co-isolated using the CTC kit. For tdEVs, the CK-PE mean intensity was plotted against the DAPI mean intensity; for edEVs, the CD105-PE mean intensity was plotted against the DAPI mean intensity. For ldEVs and leukocytes, the CD45-APC mean intensity was plotted against the DAPI mean intensity. Next to each scatter plot, four images of objects falling in each gate are shown, and the position of each image in the scatter plot is indicated with numbers 1 through 4. Nucleated cells for which the lineage of origin could not be determined, due to a lack of CD45 and cytokeratin (CK)/endoglin (CD105) expression, fall on the *y*-axis of panels A–D.

### 3.2. Estimated Size Distribution of edEVs and tdEVs Derived from Fluorescence Images

**Figure 2:**
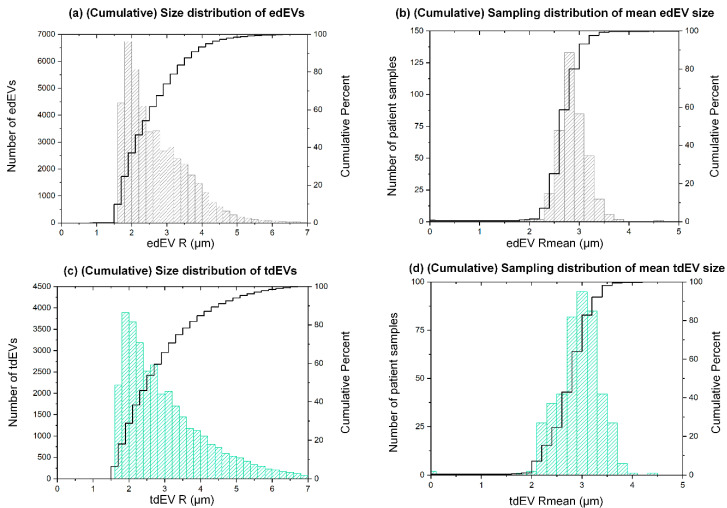
Size distribution of edEVs (**a**) and tdEVs (**c**) detected in 395 patient samples, derived from the respective CellSearch fluorescence images using the ACCEPT software. Sampling distributions of the mean edEV (**b**) and tdEV (**d**) size of 395 patient samples. The black lines correspond to the respective cumulative distributions.

The size of edEVs and tdEVs was estimated from the fluorescence images. More specifically, the PE area of each thumbnail falling in the edEV and tdEV gates (corresponding to CD105 and CKs, respectively) was measured and exported by ACCEPT. Subsequently, the size distribution plots of the herein-reported edEV and tdEV populations were constructed (Figure 2a,c). The two size distributions, even if they seem to be very similar, were significantly different (Mann–Whitney U test, *p* < 0.001), with tdEVs being slightly larger overall. The median radius of tdEVs detected in the fluorescence image datasets of 395 patient samples was 2.7 μm (interquartile range (IQR) = 1.5), with a minimum of 1.0 and a maximum of 6.9 μm. The median radius of edEVs was 2.5 μm (IQR = 1.2), with a minimum of 0.9 and maximum of 6.9 μm. Since some patient samples contributed more to these size distributions than others, as they contained more EVs, the respective sampling distributions were plotted, showing the distribution of the mean size of edEVs and tdEVs (Figure 2b,d). 80% of patient samples had an average edEV and tdEV radius smaller than 3 μm. The sampling distributions of the minimum and maximum sizes of edEVs and tdEVs is shown in Figure S1.

It is important to point out that even if the available fluorescence images allow for the detection of EVs, conclusions regarding their size cannot be unquestionable, due to the large pixel size (0.64 × 0.64 μm^2^) and the consequent lost details in the imaged objects.

### 3.3. Frequencies of CTCs, tdEVs, CECs, and edEVs in Colorectal Cancer Patients before the Initiation of a New Therapy

**Figure 3:**
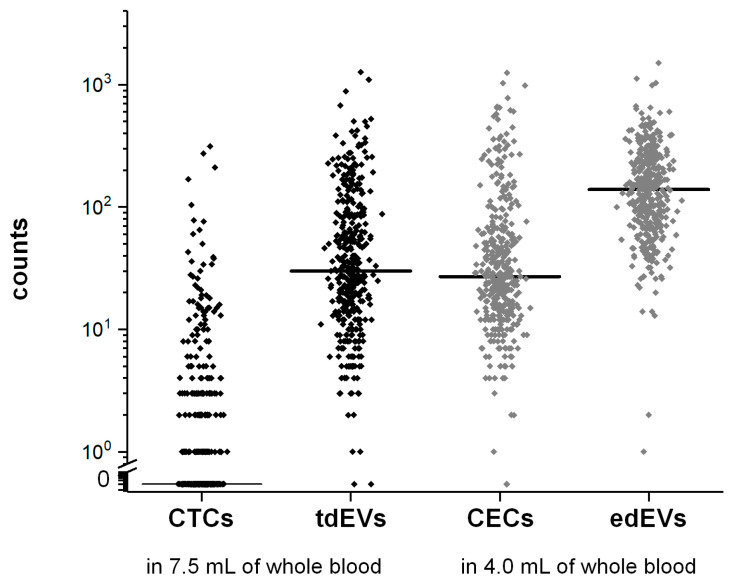
Frequencies of CellSearch isolated CTCs and tdEVs (in black) from 7.5 mL of blood, and CECs and edEVs (in grey) from 4.0 mL of blood of 395 mCRC patients before the initiation of a new therapy. The horizontal lines correspond to median values.

CTCs, tdEVs, CECs, and edEVs were enumerated in 395 mCRC patients after immunomagnetic enrichment using the CellSearch system. The results are summarized in Figure 3. Counts of CTCs ranged from 0 to 312 (median = 0, interquartile range (IQR) = 3), of tdEVs from 0 to 1268 (median = 30, IQR = 71), of CECs from 0 to 1250 (median = 27, IQR = 45), and of edEVs from 1 to 1504 (median = 139, IQR = 156).

In addition to tdEVs and edEVs, nucleated cells of unknown cell lineage of origin, CD45-expressing leukocytes, and ldEVs were identified and enumerated from the image sets generated after processing the blood samples with the CTC and CEC kits. In case of samples processed with the CEC kit, a portion of isolated leukocytes co-expressed CD45 and CD105, and were most likely monocytes in their differentiation transition to macrophages [14,15]. To facilitate the comparison of the frequencies of the co-isolated events between the two different kits used, all counts were normalized to the initial blood volume processed (7.5 mL for samples processed with the CTC kit and 4.0 mL for samples processed with the CEC kit), and the results are shown in Figure S2. Noteworthy is the observation that in contrast to tdEVs and edEVs, which appear in significantly higher frequencies than CTCs and CECs, respectively (*p* < 0.01, Wilcoxon signed ranks test), the number of ldEVs is lower than leukocytes in samples processed with either the CTC or CEC kits. The number of leukocytes and nucleated events isolated with the CEC kit are significantly higher when compared to the number of leukocytes and nucleated events isolated with the CTC kit (*p* < 0.01, Wilcoxon signed ranks test). In the case of the leukocytes, this observation can be attributed to the expression of CD146 on a subset of activated T-lymphocytes and natural killer (NK) cells, which are enriched with the CD146 ferrofluid aimed to enrich for endothelial cells [16,17,18]. In order to evaluate whether the leukocyte carryover is relative to the total leukocytes of the patients, as counted by a complete blood count (CBC), the Spearman’s Rho correlation coefficients ρ between all different CBC-based parameters and CTC and CEC isolated events were estimated. The results are summarized in Table S2. Even if many correlations were significant, most of them were very weak. Only CTCs were strongly associated with tdEVs.

### 3.4. Association of CTCs, tdEVs, CECs, and edEVs with Progression-Free and Overall Survival of Colorectal Cancer Patients before the Initiation of a New Therapy

To determine whether CECs and edEVs associate with PFS and OS, the HRs (95% CI) were calculated over a wide range of cut-off values for the CECs and edEVs of 395 patients, as illustrated in Figure S3. Only 1.8% of the possible CEC cut-off values can significantly dichotomize patients based on the HR for PFS (Figure S3A), and none of these values can result in a patient dichotomization with a significant HR for OS. On the contrary (Figure S3B), significant HRs were obtained over a wide range of edEV values (76.9% and 69.8% of all possible cutoff points, based on their PFS and OS, respectively). The optimal cut-off value of edEVs was defined as the one leading to the dichotomization of the patients with the most significant split (minimum *p*-value, log–rank) based on their HR for OS, which was found to be 287.

**Figure 4:**
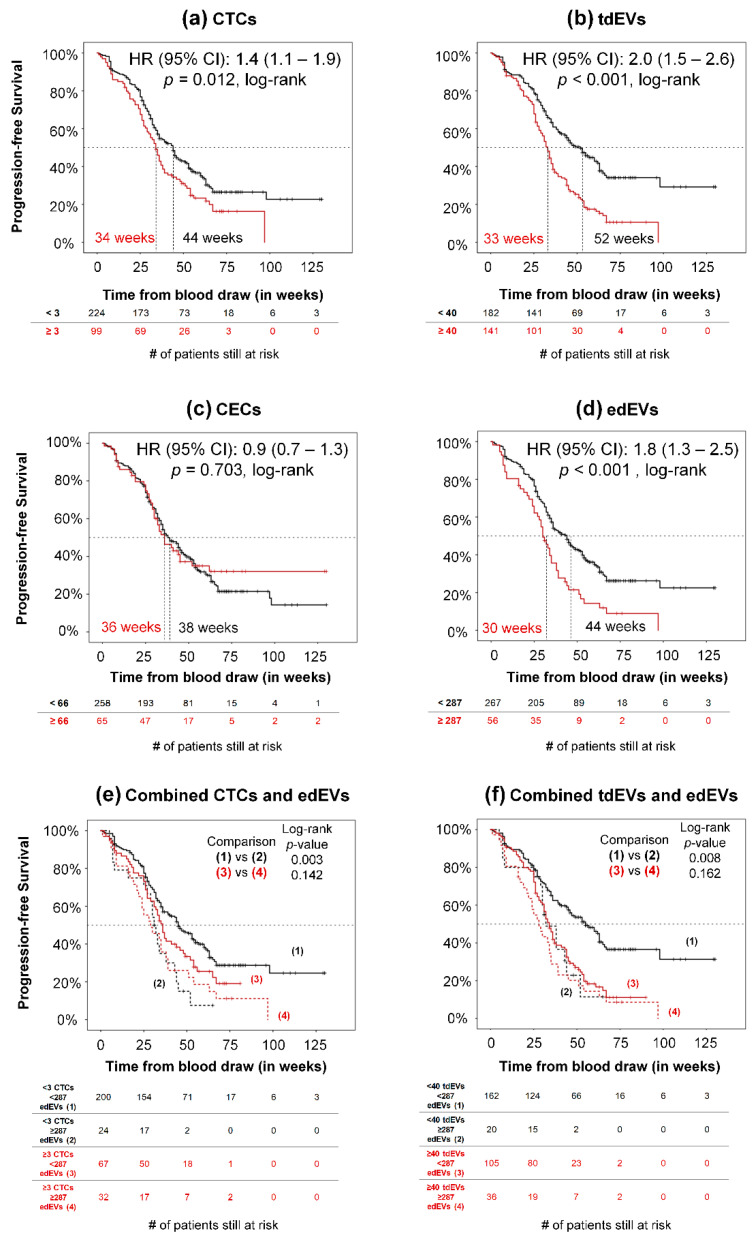
Association of CTCs (**a**), tdEVs (**b**), CECs (**c**), edEVs (**d**), combined CTCs and edEVs (**e**), and combined tdEVs and edEVs (**f**) with progression-free survival (PFS) of 323 metastatic colorectal cancer (mCRC) patients before the initiation of a new therapy.

**Figure 5:**
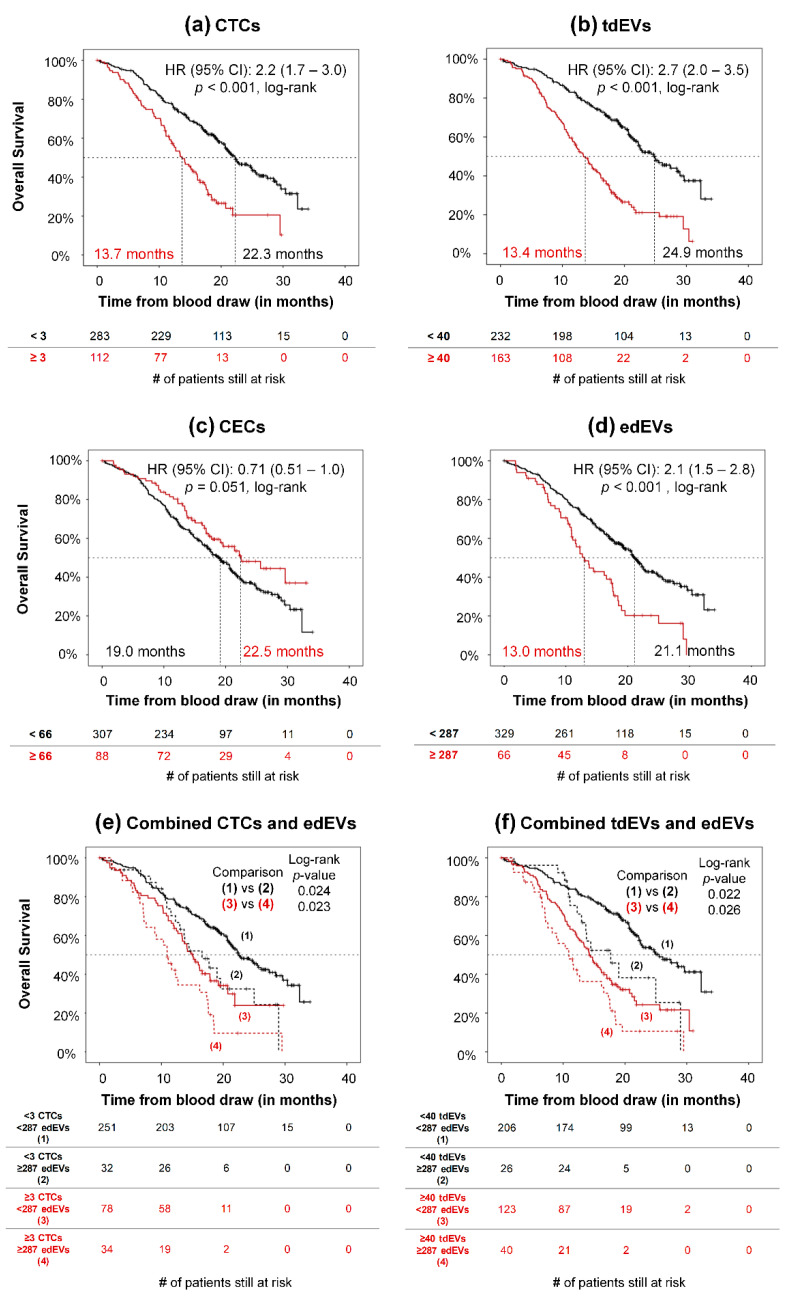
Association of CTCs (**a**), tdEVs (**b**), CECs (**c**), edEVs (**d**), combined CTCs and edEVs (**e**), and combined tdEVs and edEVs (**f**) with overall survival of 395 mCRC patients before the initiation of a new therapy.

Association of CTCs with PFS and OS and of tdEVs with OS has been reported previously [1,2,5]. Here, we demonstrate the association of PFS (Figure 4) and OS (Figure 5) of 395 mCRC patients with CTCs (a), tdEVs (b), CECs (c), and edEVs (d), generating the respective Kaplan–Meier survival plots. Patients were dichotomized using a cut-off of three or more CTCs, 40 or more tdEVs, 66 or more CECs (vertical line, Figure S3A), and 287 or more edEVs (vertical line, Figure S3B). It is clear that next to CTCs and tdEVs, elevated edEVs (but not CECs) are associated with relatively worse PFS and OS. To evaluate whether edEVs could aid in the prognostication of mCRC patients with favorable and unfavorable CTC and tdEV counts, we generated ROC curves for CTCs, tdEVs, and edEVs, as well as the combined use of CTCs or tdEVs with edEVs (Figure S4), using as a classification variable the survival of patients dichotomized by the median OS. The AUC of the combined use of CTCs or tdEVs and edEVs was significantly higher (*p <* 0.05; DeLong) compared to the AUC of only one biomarker. Kaplan–Meier survival curves of PFS and OS were generated, stratifying patients into four different risk groups after combining their edEV and CTC (Figure 4e and Figure 5e) or edEV and tdEV (Figure 4f and Figure 5f) counts. Importantly, patients with unfavorable edEV counts and favorable CTC or tdEV counts had significantly shorter PFS and OS compared to patients with both favorable edEV and CTC or tdEV counts. In case of patients with unfavorable CTC or tdEV counts, edEV counts can further stratify patients only with respect to OS.

### 3.5. Univariable and Multivariable Associations between Potential Risk Factors and Clinical Outcome (PFS and OS) of mCRC Patients

All parameters, including patient characteristics (age; gender; Eastern Cooperative Oncology Group (ECOG) performance status; prior adjuvant therapy; number of metastatic sites; primary tumor in situ; sidedness of primary tumor; presence of KRAS, NRAS, and BRAF mutations; treatment arm), baseline serum lactate dehydrogenase (LDH), alkaline phosphatase (ALP), CBC-based parameters (absolute leukocyte counts, absolute platelet counts, hemoglobin), and all different classes isolated with the CTC and CEC kits (CTCs, tdEVs, CECs, edEVs, leukocytes, nucleated events, ldEVs, CD105+ leukocytes, and CD105+ ldEVs) were evaluated as potential risk factors of PFS and OS, using univariable Cox proportional hazards regression analysis. Next to the continuous log-transformed CTCs, tdEVs, CECs, and edEVs, the dichotomized CTCs, tdEVs, CECs and edEVs were also included in the analysis and are shown in Table 1. Surprisingly, leukocytes present in the cartridges after EpCAM enrichment showed a significant association with PFS and OS; the nucleated events after EpCAM enrichment were also significantly associated with OS (not PFS). In contrast to EpCAM-enriched leukocytes and nucleated events, the respective populations after CD146 enrichment were not significantly associated with worse PFS or OS.

For the multivariable analysis, the significant predictors from the univariable analysis were included. The number of metastatic sites, presence of BRAF mutation, and hemoglobin remained significant predictors of PFS in the final multivariable model (Table 2). The number of metastatic sites, presence of BRAF mutation, right-sidedness of the primary tumor, platelets (log-transformed), and tdEVs (log-transformed) remained significant predictors of OS in the final multivariable model (Table 2).

### 3.6. Association of Clinical Parameters with tdEVs, CTCs, CECs and edEVs

We evaluated the strength of association between clinical parameters and the biomarkers under investigation, namely CTCs, tdEVs, CECs, and edEVs, using the non-parametric Spearman’s Rho test. The results are summarized in Table S3. CECs were correlated significantly only with hemoglobin and prior adjuvant therapy, but the strength of that association was very weak (ρ < 0.2). Even if many clinical parameters were significantly correlated with edEVs, tdEVs, and CTCs, most associations were (very) weak (ρ < 0.4). Interestingly, CTCs and tdEVs had a significant association of moderate strength (0.4 < ρ < 0.6) with the LDH and ALP serum values.

## 4. Discussion

Liquid biopsies have attracted the interest of many research groups thanks to their potential to improve the disease management of cancer patients in a non-invasive and timely fashion. Circulating tumor cells have emerged as significant predictors of overall survival of castration-resistant prostate, metastatic breast, metastatic colorectal, and non-small-cell lung cancer patients [1,4,19,20]. Importantly, their phenotypic and genotypic characterization can shed light into new therapeutic targets and predict the treatments that will lead to the recession of specific patient groups [21,22]. However, the scarceness of CTCs in the blood, their frequently apoptotic status, and the leukocyte carryover after the chosen enrichment technique impedes their downstream genetic analysis [23,24]. Therefore, complementary research is being conducted towards the discovery of additional biomarkers predictive of treatment benefit.

Large, tumor-derived extracellular vesicles isolated together with CTCs from blood samples of metastatic prostate, breast, colorectal, and non-small cell lung cancer (NSCLC) patients are present in significantly higher frequencies than CTCs, while having an equivalent prognostic power to them [3,4,5]. Furthermore, they seem to better reflect the phenotype of the primary tumor compared to CTCs [25]. Studies of Di Vizio et al. support that large oncosomes with similar characteristics as the tdEVs we have reported in the present study (in regards to their size and expression of CKs and EpCAM), are released by non-apoptotic and highly aggressive amoeboid tumor cells [26]. Interestingly, we observed that CTCs and tdEVs have a significant association of moderate strength with the serum LDH and ALP values of mCRC patients. Elevated LDH serum levels of cancer patients have been linked to tumor hypoxia and worse clinical outcome [27,28,29,30]. It was previously shown that hypoxia stimulates the upregulation of tumor progression genes in cancer cell lines [31]. In alignment with these findings, Kallergi et al. demonstrated the expression of hypoxia-inducible factor-1α in around 60% of the total CTCs of around 75% of breast cancer patients [32]. Furthermore, increased ALP is an indicator of tumor invasiveness, since it is associated with bone and liver metastases. All these observations together support the hypothesis that hypoxia is a driving force of tumor aggressiveness, manifested by increased CTCs and tdEVs in the blood circulation of cancer patients.

Circulating endothelial cells hold great promise, since their elevated counts could be an indicator of tumor angiogenesis or vascular damage due to the tumor growth; thus, they could predict patient response to anti-angiogenic treatments. A variety of assays for CEC enumeration have been reported; however, the lack of a consensus on the CEC phenotype and of standardized techniques for CEC isolation and detection hinders the comparison of results between different studies and the drawing of incontrovertible conclusions [33]. The CEC assay that we used in the present study, immunomagnetically enriched CD146+ CECs from 4 mL of whole blood and identified CECs based on presence of nucleus, expression of CD105, and lack of CD45. CEC frequencies in blood of cancer patients are elevated compared to the CEC frequencies in healthy donors [6]. However, no association with clinical outcome could be observed in metastatic prostate cancer [34] and mCRC [7]. Our results confirm these findings for all possible cut-off values of CECs. One explanation is that CEC counts are biased due to the endothelial cells being detached during the blood draw [6]. A solution to eliminate that bias could be the use of antibodies targeting exclusively cancer-associated CECs [35]. However, in a study in which CECs were defined as DAPI+, CD34+, CD146+, and CD45−, and also expressed the immune check molecule CD276, which is frequently associated with cancer, no association could be found with poor outcome or treatment responses [36].

As tdEVs can be detected in blood samples enriched for CTCs, we asked ourselves whether edEVs could also be detected in blood samples enriched for CECs, and whether their presence is associated with clinical outcome. In contrast to CEC counts, which are influenced by a portion of endothelial cells originating from the vascular wall due to the blood draw, the edEV count is expected to be less biased.

Previous studies have reported the presence of edEVs in different pathophysiological conditions, as analyzed mainly with flow cytometry [37,38,39]. However, the need for a standardized isolation technique and a consensus on their definition has been stressed in several studies [40,41]. Duval et al. compared different antibody combinations using flow cytometry, and concluded that the CD146+, CD105+, and CD45- definition results in the most efficient detection of the edEV population [37]. Consistent with their probing, in our study CD146 was used to immunomagnetically enrich for CECs and edEVs, CD105 was used as an inclusion marker for their detection, and CD45 was an exclusion marker to discriminate them from the leukocyte carryover. We were able to detect edEVs, but the large pixel size of the acquired fluorescence images (0.64 × 0.64 μm^2^) let us estimate rather than accurately determine their size distribution (Figure 2). Since the smallest detected EVs in the present study, based on the immunofluorescence images, had a radius of 1 μm, questions arise regarding their biogenesis. What portion of the CellSearch isolated edEVs (and tdEVs) are apoptotic bodies and apoptotic microvesicles [42] remains to be addressed in a prospective study by the inclusion of an additional marker for apoptosis in the CellSearch runs, such as the apotracker or annexin V. A better classification of the herein-reported EVs into microvesicles and apoptotic bodies could lead to more selective biomarkers for the evaluation of the clinical outcome of the patients and their response to treatments. Moreover, the detection and further classification of edEVs and tdEVs in the plasma fraction should be explored further, as it is likely that the majority of edEVs and tdEVs will reside in this fraction. Whether or not they will have a similar relation with clinical outcome or encompass a different class of edEVs and tdEVs will have to be investigated.

Importantly, when plotting all possible cut-off values of edEV counts versus the HR, more than half of the values could significantly dichotomize the patients into more and less favorable groups of PFS (76.9% of cut-off values) and OS (69.8% of cut-off values) (Figure S3). This was into contrast with CECs, for which only 1.8% of possible cut-off values were significant in the case of only PFS. The cutoff value of 287 edEVs in 4 mL of peripheral blood was chosen as the value leading to the most significant split of the patients. The group of patients with elevated edEVs experienced significantly shorter median PFS and OS (Figure 3 and Figure 4) compared to the patients with <287 edEVs.

The weak association between edEVs and tdEVs (Table S3) suggests partially independent underlying biological functions of edEVs and tdEVs in the metastatic process of colorectal cancer. There is not much data on the biological functions of large edEVs. However, Hristov et al. reported that edEVs originated from apoptotic endothelial cells enhance the differentiation of endothelial progenitors [43]. In case of cancer, that differentiation could lead to increased angiogenesis at the tumor sites. In the present study, patients of both treatment arms received the angiogenesis inhibitor bevacizumab, which does not allow us to determine a possible effect of the drug on the edEV release. No difference in the edEV counts could be detected in patients in which the epidermal growth factor receptor (EGFR) inhibitor cetuximab was administered or not (data not shown).

Last but not least, we unexpectedly noticed that mCRC patients with increased counts of leukocytes co-isolated after EpCAM enrichment had significantly worse OS and PFS (Table 1). In contrast, no association with clinical outcome could be found between the carried-over leukocytes using the CD146 enrichment (Table 1). Previously, we reported the presence of a significantly larger number of leukocytes and nucleated cells of unknown origin after EpCAM enrichment in blood of NSCLC patients, compared to healthy donors [43]. Up to now, leukocytes detected in the CellSearch cartridges were considered to constitute a non-specific carryover. The weak correlation between the leukocyte counts in the EpCAM-enriched samples and the leukocyte counts from the available complete blood counts (Table S1) rejects a plausible explanation that the leukocyte carryover reflects the total leukocyte count of patients, with the latter known to be associated with patients’ clinical outcome in bladder, prostate, and non-small-cell lung cancers [44,45,46,47], as well as in colorectal cancer (Table 1). That observation raised questions about the subtype of leukocytes isolated with the CTC kit, which either overexpress Fcγ receptors that bind to the IgG immunoglobulins of the anti-EpCAM ferrofluid, or express EpCAM. Fc receptors are expressed on all hematopoietic cells, playing a key role in immune modulation, having both inhibitory and activating functions [48,49]. The increased expression of Fcγ receptors on the monocytes of gastric cancer patients has been previously reported [50]. An alternative plausible explanation would be the expression of EpCAM on a subset of leukocytes and their specific isolation using the CTC kit. EpCAM belongs in the CAM family, serving a diversity of cell functions [51]. Its expression is known to be restricted only in epithelial cells in healthy humans. *De novo* expression of EpCAM has been reported in mature human hepatocytes during various inflammatory liver diseases and liver regeneration and repair [52,53]. Furthermore, EpCAM is expressed also by a subset of thymocytes, as well as B and T lymphocytes in mice, facilitating the cells’ infiltration in inflammation sites [54,55]. Similarly, the upregulation of integrins, which comprise a CAM family, by effector T lymphocytes also facilitate their targeted access to inflammatory sites [56]. However, to our knowledge, it has not been investigated whereas EpCAM is expressed in any subsets of leukocytes in cancer patients that would facilitate the leukocytes’ infiltration in the tumor. The EpCAM expression on the surfaces of the leukocyte could emanate from (a) the activation of originally silent molecular pathways, (b) the uptake of tumor-derived extracellular vesicles by cell–EV membrane fusion [57], or (c) the internalization and expression of functional EpCAM mRNA by leukocytes [58]. The two latter suggestions are supported by the findings that tdEVs accumulate in all leukocyte subpopulations in murine models, altering the phenotypes of the recipient cells [59,60].

## 5. Conclusions

Endothelium-derived extracellular vesicles, isolated based on their CD146 expression and detected based on their CD105 expression, are significant predictors of PFS and OS in metastatic colorectal cancer, in contrast to circulating endothelial cells. Interestingly, the EpCAM-based co-isolated leukocytes constitute a significant predictor of OS and PFS, suggesting further investigation on the phenotype of that population.

## Figures and Tables

**Table 1:** Univariable Cox proportional hazards regression analysis.

Variable	Cut-Off	PFS Risk		OS Risk		
		HR (95% CI)	*p*-Value	HR (95% CI)	*p*-Value	
Prior adjuvant therapy		0.7 (0.4–1.0)	0.043	0.7 (0.4–1.0)	0.046	
Number of metastatic sites		1.3 (1.2–1.5)	<0.001	1.3 (1.2–1.5)	< 0.001	
Primary tumor in situ		1.5 (1.1–2.0)	0.013	1.8 (1.3–2.4)	< 0.001	
Presence of KRAS mutation		1.0 (0.7–1.3)	0.839	1.1 (0.8–1.5)	0.666	
Presence of BRAF mutation		3.0 (2.0–4.6)	<0.001	2.7 (1.8–4.0)	< 0.001	
Presence of NRAS mutation		1.19 (0.60–2.40)	0.629	1.0 (0.5–2.0)	1.000	
Right-sidedness of primary tumor (transverse colon, hepatic flexure, ascending colon, cecum)		1.3 (1.0–1.8)	0.042	1.6 (1.2–2.1)	0.001	
Treatment arm		1.1 (0.9–1.4)	0.465	1.2 (0.9–1.5)	0.313	
ECOG performance status		1.4 (1.1–1.8)	0.012	1.4 (1.1–1.9)	0.006	
age		1.0 (1.0–1.0)	0.078	1.0 (1.0–1.0)	0.290	
gender		1.0 (0.7–1.3)	0.958	1.0 (0.7–1.3)	0.773	
**CellSearch Populations**						
CTCs (log transformed)	-	1.2 (1.1–1.4)	0.004	1.6 (1.4–1.9)	<0.001	
≥ 3 CTCs	3	1.4 (1.1–1.9)	0.012	2.2 (1.7–3.0)	<0.001	
tdEVs (log-transformed)	-	1.8 (1.4–2.3)	<0.001	2.5 (2.0–3.2)	<0.001	
≥ 40 tdEVs	40	2.0 (1.5–2.6)	<0.001	2.7 (2.0–3.5)	<0.001	
CECs (log-transformed)	-	1.1 (0.9–1.5)	0.338	1.0 (0.8–1.2)	0.767	
≥ 66 CECs	66	0.9 (0.7–1.3)	0.703	0.7 (0.5–1.0)	0.051	
edEVs (log-transformed)	-	1.7 (1.2–2.5)	0.004	1.9 (1.3–2.8)	0.002	
≥ 287 edEVs	287	1.8 (1.3–2.5)	<0.001	2.1 (1.5–2.8)	<0.001	
**Other CellSearch Populations**						
Leukocytes ^a^ (log-transformed)	-	1.3 (1.1–1.5)	0.009	1.3 (1.1–1.6)	0.001	
ldEVs ^a^ (log-transformed)	-	1.1 (0.9–1.4)	0.437	1.2 (0.9–1.5)	0.219	
Nucleated ^a^ (log-transformed)	-	1.1 (0.9–1.4)	0.239	1.3 (1.0–1.6)	0.048	
Leukocytes ^b^ (log-transformed)	-	1.1 (0.8–1.5)	0.737	1.1 (0.8–1.5)	0.751	
ldEVs ^b^ (log-transformed)	-	1.0 (0.7–1.4)	0.976	1.1 (0.8–1.6)	0.608	
Nucleated ^b^ (log-transformed)	-	1.1 (0.9–1.5)	0.436	1.3 (1.0–1.7)	0.091	
CD105+ leukocytes ^b^ (log-transformed)	-	1.0 (0.9–1.2)	0.789	1.0 (0.8–1.2)	0.927	
CD105+ ldEVs ^b^ (log-transformed)	-	1.0 (0.8–1.2)	0.842	1.1 (0.9–1.4)	0.203	
**CBC-Based Parameters**						
Leukocytes (log-transformed)	-	6.0 (2.1–16.9)	0.001	9.5 (3.5–25.9)	<0.001	
Platelets (log-transformed)	-	4.0 (1.6–10.3)	0.004	5.3 (2.1–13.4)	<0.001	
Hemoglobin, per 1 g/dL	-	0.8 (0.7–0.9)	<0.001	0.7 (0.6–0.8)	<0.001	
Alkaline phosphatase (ALP) (log-transformed)		2.7 (1.6–4.4)	<0.001	3.8 (2.4–6.2)	<0.001	
Baseline serum lactate Dehydrogenase (LDH) (log-transformed)		1.9 (1.3–2.8)	0.002	2.4 (1.6–3.5)	<0.001	

^a^ Refers to populations co-isolated with the CTC kit; ^b^ refers to populations co-isolated with the circulating endothelial cell (CEC) kit.

**Table 2:** Multivariable Cox proportional hazards regression analysis (using backward and forward stepwise elimination).

Variable in Equation	PFS Risk		OS Risk	
	HR (95% CI)	*p*-Value	HR (95% CI)	*p*-Value
Presence of BRAF mutation	2.8 (1.8–4.4)	< 0.001	2.3 (1.4–3.6)	<0.001
tdEVs (log-transformed)			1.8 (1.3–2.4)	0.001
Number of metastatic sites	1.4 (1.2–1.7)	< 0.001	1.3 (1.1–1.5)	0.003
Right-sidedness of the primary tumor			1.6 (1.1–2.3)	0.010
Platelets (log transformed)			4.6 (1.4–15.1)	0.013
Hemoglobin, per 1 g/dL	0.8 (0.7–1.0)	0.048		

## Supplementary Materials

The following are available online at https://www.mdpi.com/2073-4409/9/12/2688/s1, Figure S1: Sampling distributions of mininum edEV size (a), maximum edEV size (b), minimum tdEV size (c), and maximum tdEV size (d). The black lines in each graph correspond to the respective cumulative distributions. Figure S2: Frequencies of different populations co-isolated with the CTC (black dots) and CEC kits (grey dots), normalized to 1 mL of blood for comparison between the kits. Figure S3: Cut-off optimization of the baseline values for CECs (A) and edEVs (B) in mCRC patients. For each possible cut-off, CECs and edEVs were correlated with PFS (top) or overall survival (OS) (bottom). The hazard ratio (HR), including 95% CI, was plotted depending on the cutoff. The vertical line indicates the cut-off that results in the most significant correlation with OS. The value distribution of CECs and edEVs is shown as a rug plot at the bottom of the respective figure. Figure S4: Receiver operating characteristic (ROC) curves treating survival time dichotomized by the median OS time of the patient cohort as the classification variable. The addition of edEVs to CTCs (A) or tdEVs (B) results in a significantly (*p* < 0.05) higher area under the curve (AUC) compared to solely CTCs or tdEVs. Table S1: ACCEPT gates for the automated enumeration of tdEVs, edEVs, ldEVs, leukocytes, and nucleated events. The exponents ^a^ and ^b^ correspond to the CTC and CEC kit, respectively. Table S2: Correlation between CBC-based parameters, CTC and CEC kit-isolated objects of 395 mCRC patients, using the Spearman’s Rho correlation coefficient (ρ). Correlation is considered to be weak at ρ < 0.4 (in grey), moderate at 0.4 ≤ ρ <0.6 (in black), and strong at 0.6 ≤ ρ ≤0.8 (in bold black). * Indicates significance at 0.05 level (two-tailed); ** indicates significance at 0.01 level (two-tailed). Table S3: Correlation between clinical parameters and CTCs, tdEVs, CECs, and edEVs of 395 mCRC patients using the Spearman’s Rho correlation coefficient (ρ). Correlation is considered to be weak at ρ < 0.4 (in grey), moderate at 0.4 ≤ ρ <0.6 (in black), and strong at 0.6 ≤ ρ ≤0.8 (in bold black). ** Indicates significance at 0.01 level (two-tailed); * indicates significance at 0.05 level (two-tailed).
