# Supplementary material for: Endothelium-Derived Extracellular Vesicles Associate with Poor Prognosis in Metastatic Colorectal Cancer
Source: Cells. 2020 Dec 15;9(12):2688. doi: 10.3390/cells9122688 (PMC7765205; doi:10.3390/cells9122688)
Supplement: Supplementary file 1 [file cells-09-02688-s001.pdf]

cells

MDPI

Article

Endothelium-Derived Extracellular Vesicles Associated with Poor Prognosis in Metastatic Colorectal Cancer

Afroditi Nanou [1,*], Linda Mol [2], Frank A.W. Coumans [1], Miriam Koopman [3], Cornelis J.A. Punt [4] and Leon W.M.M. Terstappen [1,*]

1. Department of Medical Cell BioPhysics, University of Twente, 7522ND Enschede, The Netherlands; f.a.w.coumans@utwnte.nl
2. Netherlands Comprehensive Cancer Organization, 6533AA Nijmegen, The Netherlands; L.Mol@iknl.nl
3. Department of Medical Oncology, University Medical Center Utrecht, 3584CS Utrecht, The Netherlands; m.koopman-6@umcutrecht.nl
4. Department of Epidemiology, Julius Center for Health Sciences and Primary Care, University Medical Center Utrecht, 3584CG Utrecht, The Netherlands; C.J.A.Punt@umcutrecht.nl

* Correspondence: a.nanou@utwente.nl (A.N); l.w.m.m.terstappen@utwente.nl (L.W.M.M.T); Tel.: +31-613-626-229 (A.N)

Received: 23 October 2020; Accepted: 11 December 2020; Published: 15 December 2020

Table S1. ACCEPT gates for the automated enumeration of tdEVs, edEVs, ldEVs, leukocytes and nucleated events.

Gates\Marker	DNA[a,b]	CD45 [a,b]		CK [a]/CD105 [b]						Marker1 [a,b]
	Mean Intensity	Mean Intensity		Mean Intensity	Max Intensity	Perimeter (in pixels)	Eccentricity	Size (in µm[2])	Perimeter/Area	Mean Intensity
tdEVs[a]	≤5	≤5		>60	>90	>5	≤0.8	≤150	≤1	≤5
edEVs[b]	≤5	≤5		>40	>60	>5	≤0.8	≤150	≤1	≤5
	DNA [a,b]	CK [a]/CD105 [b]		CD45 [a,b]						Marker1 [a,b]
	Mean Intensity	Mean Intensity	Overlay with DNA	Mean Intensity	Max Intensity	Perimeter (in pixels)	Eccentricity	Size (in µm[2])	Perimeter/Area	Mean Intensity
ldEVs[a,b]	≤5	≤5	-	>30	>50	>5	≤0.8	≤150	≤1	≤5
leukocytes[a,b]	>30	≤5	-	>30	>50	-	-	>16	-	≤5
Nucleated[a,b]	>30	≤5	-	≤5	-	-	-	>16	-	≤5
CD105+ ldEVs[b]	≤5	>40	-	>30	>50	>5	≤0.8	≤150	≤1	≤5
CD105+ leukocytes[b]	>30	>40	>0.2	>30	>50	-	-	>16	-	≤5

The exponents a and b correspond to the CTC and CEC kit, respectively.

Table S2. Correlation between CBC-based parameters, CTC- and CEC- kit isolated objects of 395 mCRC patients using the Spearman's Rho correlation coefficient (ϱ). Correlation is considered to be weak for ϱ < 0.4 (in grey), moderate for 0.4 ≤ ϱ <0.6 (in black) and strong for 0.6 ≤ ϱ ≤0.8 (in bold black).

	**Parameters**	**CTC kit isolated**					**CEC kit isolated**						
		**Leukocytes**	**Nucleated**	**ldEVs**	**CTCs**	**tdEVs**	**Leukocytes**	**CD105 Leukocytes**	**Nucleated**	CD105 ldEVs	ldEVs	CECs	edEVs
**CBC-based**	CBC Leukocytes	.208 **	.257**	.170 **	.276**	.234 **	.378 **	.112*	.299 **	0.023	.227 **	0.009	.238 **
	CBC hemoglobin	-0.072	-.179**	0.038	-.168**	-.167**	-0.064	-.202 **	-.204**	-.127*	-.111*	-.179 **	-.315 **
	CBC platelets	0.072	.109 *	0.037	.208**	.197**	.266 **	.151 **	.207 **	0.000	.155 **	0.075	.260 **
**CTC kit isolated**	Leukocytes	1.000	.473 **	.437 **	.160**	.200**	.172**	.184 **	.213**	0.022	-0.054	0.065	.164 **
	Nucleated		1.000	.366 **	.119*	.164 **	.140**	.212 **	.520 **	-0.079	-0.038	0.048	.190**
	ldEVs			1.000	0.036	.186 **	.196 **	.135 **	.143**	-0.024	0.062	0.069	.140**
	CTCs				1.000	**.694 ** **	-0.004	-0.012	-0.012	-0.010	0.017	0.015	.285**
	tdEVs					1.000	-0.026	0.007	-0.022	0.023	-0.020	0.023	.283**
**CEC kit isolated**	Leukocytes						1.000	.202**	.286 **	0.009	.459 **	0.033	.142**
	CD105 Leukocytes							1.000	.184 **	.218 **	-0.013	.447 **	.417 **
	Nucleated								1.000	-0.007	0.041	0.015	0.058
	CD105 ldEVs									1.000	.178**	.147 **	.291 **
	ldEVs										1.000	-0.009	.120 *
	CECs											1.000	.450 **
	edEVs												1.000

** indicates significance at 0.01 level (2-tailed), * indicates significance at 0.05 level (2-tailed).

Table S3. Correlation between clinical parameters and CTCs, tdEVs, CECs and edEVs of 395 mCRC patients using the Spearman's Rho correlation coefficient (ϱ). Correlation is considered to be weak for ϱ < 0.4 (in grey), moderate for 0.4 ≤ ϱ < 0.6 (in black) and strong for 0.6 ≤ ϱ ≤0.8 (in bold black).

Clinical parameter	edEVs	CECs	tdEVs	CTCs
Primary tumor in situ	.146 **	-0.074	.323 **	.295 **
Presence of KRAS mutation	-0.082	-0.078	-0.042	-0.077
Presence of BRAF mutation	0.086	0.078	0.003	-0.037
Presence of NRAS mutation	-0.040	0.064	-0.121	-0.059
Right sidedness of primary tumor	0.081	0.060	0.056	-0.009
Treatment arm	-0.016	-0.075	0.055	0.061
Prior adjuvant therapy	-.213 **	-.109 *	-.159 **	-.133 **
ECOG performance status	.107 *	0.020	.123 *	.118 *
gender	-0.008	0.051	-0.050	-.134 **
Number of metastatic sites	.110 *	0.019	.112 *	0.084
age	-0.043	0.045	-0.093	-.135 **
Lactate dehydrogenase (LDH)	.277 **	0.093	.458 **	.485 **
Alkaline Phosphatase (ALP)	.348 **	0.080	.463 **	.486 **
CBC leukocytes	.238 **	0.009	.234 **	.276 **
CBC hemoglobin	-.315 **	-.179 **	-.167 **	-.168 **
CBC platelets	.260 **	0.075	.197 **	.208 **

** indicates significance at 0.01 level (2-tailed), * indicates significance at 0.05 level (2-tailed).

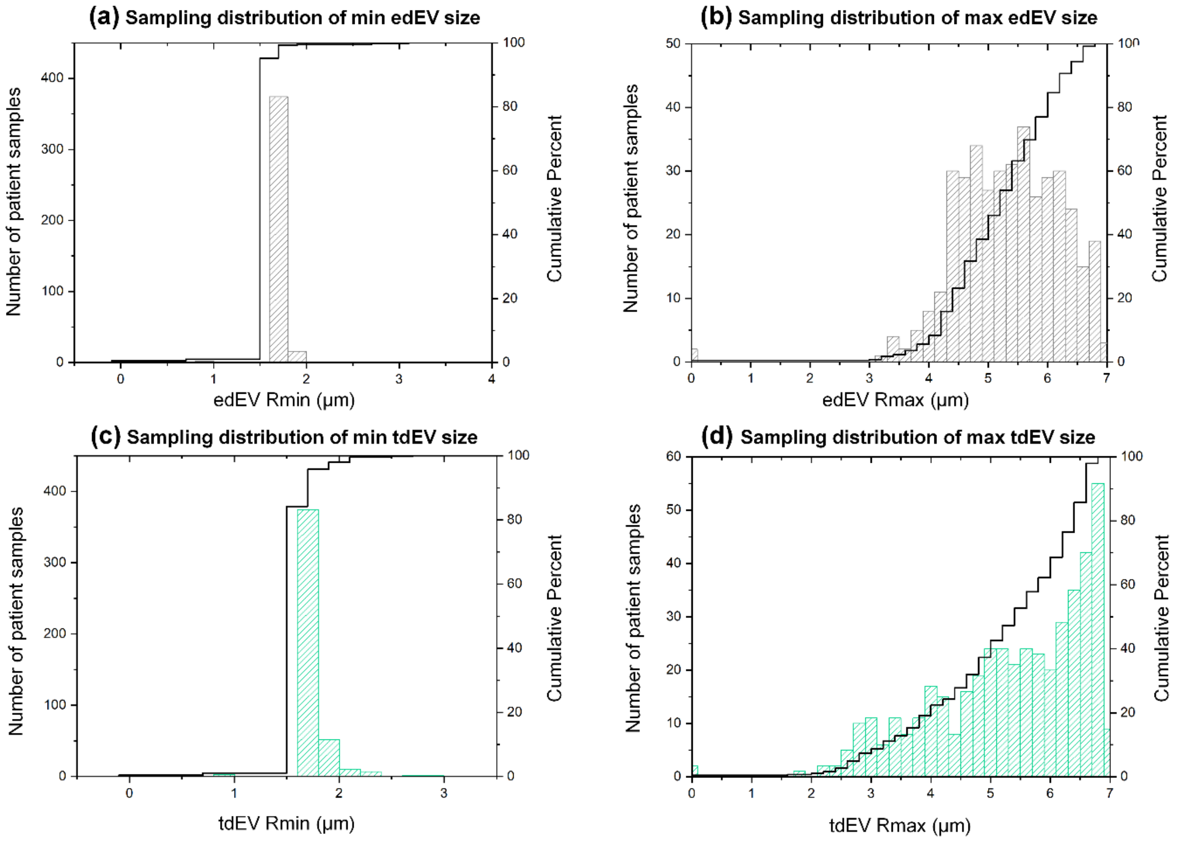

Figure S1. Sampling distributions of min edEV size (**a**), max edEV size (**b**), min tdEV size (**c**) and max tdEV size (**d**). The black lines in each graph correspond to the respective cumulative distributions.

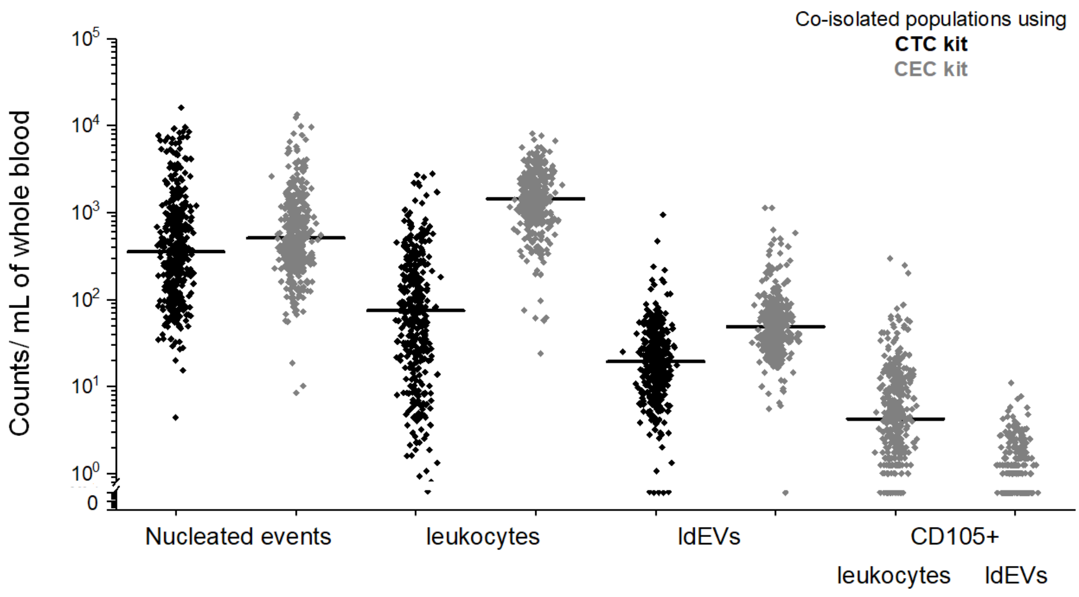

Figure S2. Frequencies of different populations co-isolated with the CTC (black dots) and CEC kits (grey dots), normalized to 1 mL of blood for comparison between the kits.

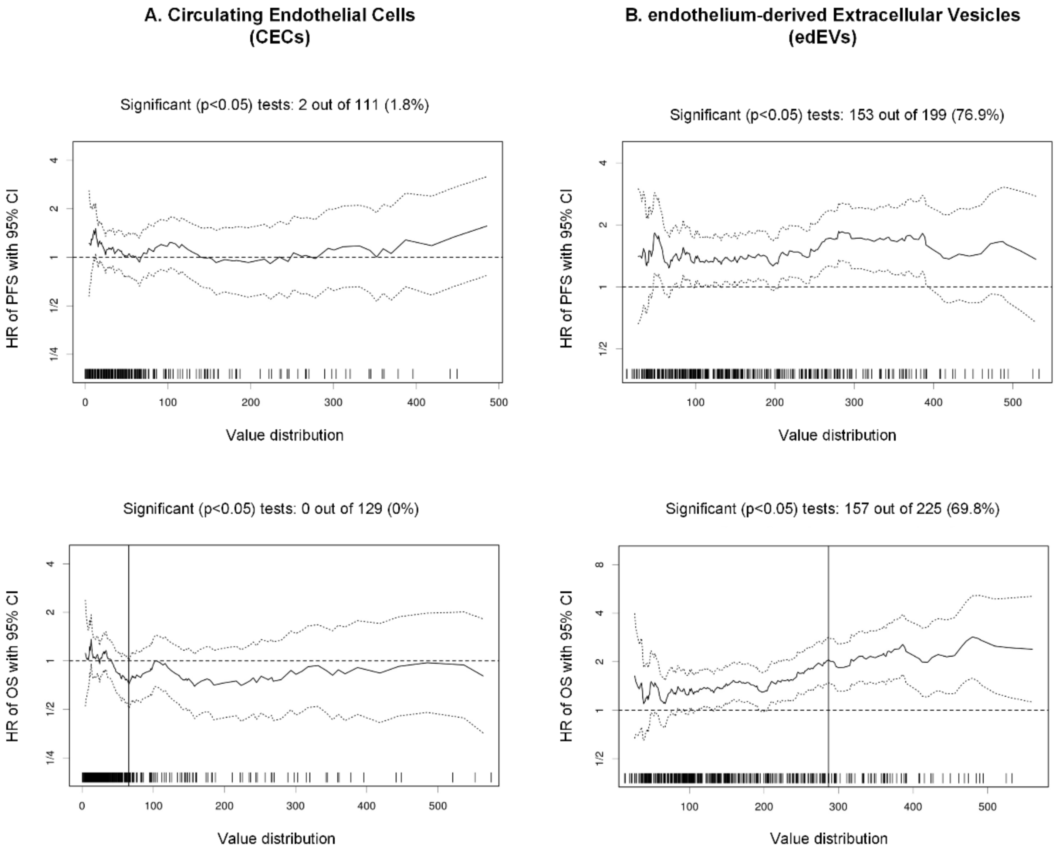

Figure S3. Cut-off optimization of the baseline values for CECs (**A**) and edEVs (**B**) in mCRC patients. For each possible cut-off, CECs and edEVs were correlated with PFS (top) or OS (bottom). The HR including 95% CI was plotted in dependence of the cutoff. The vertical line indicates the cut-off that results in the most significant correlation with OS. The value distribution of CECs and edEVs is shown as a rug plot at the bottom of the respective figure.

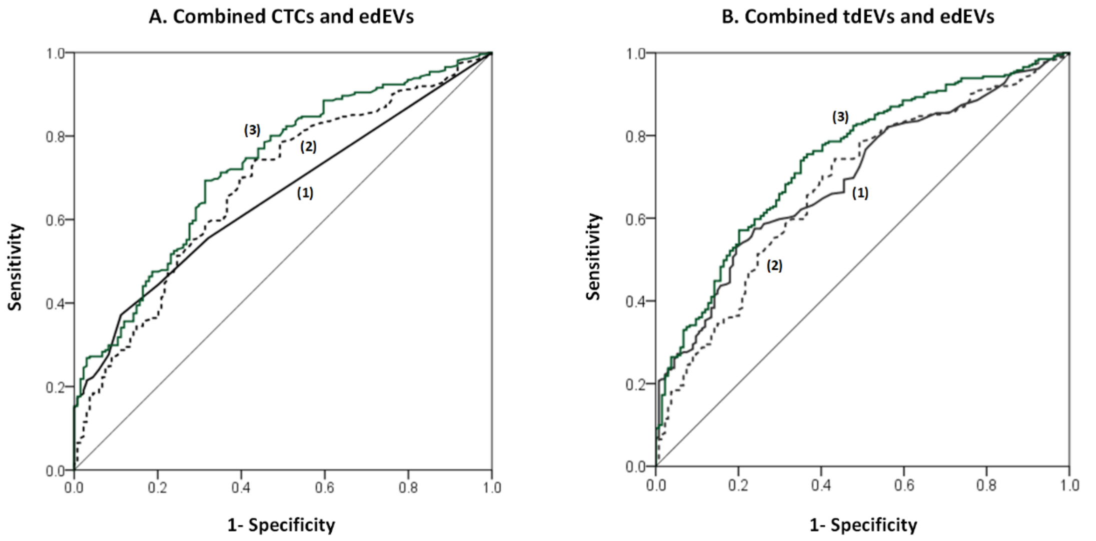

Variable	% AUC	p-value
**CTCs (1)**	65.2 (59.8 – 70.7)	< 0.001
**edEVs (2)**	67.8 (62.3 – 73.3)	< 0.001
CTCs & edEVs **(3)**	72.5 (67.3 – 77.6)	< 0.001

Variable	% AUC	p-value
**tdEVs (1)**	69.6 (64.3 – 74.8)	< 0.001
**edEVs (2)**	67.8 (62.3 – 73.3)	< 0.001
tdEVs & edEVs **(3)**	74.4 (69.4 – 79.4)	< 0.001

Figure S4. Receiver Operating Characteristic (ROC) curves treating survival time dichotomized by the median OS time of the patient cohort as the classification variable. The addition of edEVs to CTCs (**A**) or tdEVs (**B**) results in significantly ($p < 0.05$) higher area under the curve (AUC) compared to solely CTCs or tdEVs.
